# Pre-stem cell formation by non-platelet RNA-containing particle fusion

**DOI:** 10.1111/1440-1681.12101

**Published:** 2013-06-21

**Authors:** Wuyi Kong, Mu Nuo, Xiao Ping Zhu, Xiu Juan Han, Lihua Luo, Xian Wang

**Affiliations:** * Beijing Khasar Medical Technology Co.Beijing, China; † Department of Obstetrics, Beijing HospitalBeijing, China; ‡ Department of Physiology, Beijing UniversityBeijing, China

**Keywords:** non-nucleated cells, octamer-binding transcription factor 4 (OCT4), sex-determining region Y 2 (SOX2), stem cells, umbilical cord blood

## Abstract

1. We found a group of non-platelet RNA-containing particles (NPRCP) in human umbilical cord blood. To understand the origin, characterization and differentiation of NPRCP, we examined cord blood-isolated NPRCP *in vitro*.

2. The NPRCP range in size from < 1 to 5 μm, have a thin bilayer membrane and various morphological features, contain short RNA and microRNA and express octamer-binding transcription factor 4 (OCT4), sex-determining region Y 2 (SOX2) and DEAD box polypeptide 4 (DDX4). On coculture with nucleated cells from umbilical cord blood, NPRCP fuse to small, active, non-nucleated cells called ‘particle fusion-derived non-nucleated cells’ (PFDNC). The PFDNC are approximately 8 μm in diameter and are characterized by their twisting movement in culture plates. They can easily move into and out of nucleated cells and finally differentiate into mesenchymal-like cells. In addition, the larger non-nucleated cellular structures that are derived from the aggregation and fusion of multiple NPRCP can further differentiate into large stem cells that also release OCT4- and SOX2-positive non-nucleated small cells.

3. Our data provide strong evidence that NPRCP can fuse into PFDNC, which further differentiate into mesenchymal-like cells. Multiple NPRCP also fuse into other types of large stem cells. We believe that stem cells are derived from NPRCP fusion. There is considerable potential for the use of NPRCP in clinical therapy.

## Introduction

Two unsolved questions about adult stem cells are still under debate after more than a decade of studies. One is what type or types of adult stem cells possess the multilineage function? The other is whether stem cells can self-renew, which has been described as an asymmetric mitotic division.^1^ Many researchers have long believed that bone marrow (BM) and blood are the postnatal stem cell niches for tissue regeneration.^2^,^3^ Unfractionated BM and blood stem cells can regenerate muscle, neurons, hepatocytes, smooth muscle cells and other tissues^4^–^7^ via differentiation of mesenchymal stem cells (MSC). Despite many attempts to prospectively isolate multipotent MSC, most commonly by fluorescent active cell sorting, the proof for multipotent BM stem cells has been unsatisfactory.^8^ In the past decade, several stem cells with multipotent function have been reported, including: (i) multipotent adult progenitor cells, characterized as CD34^–^, CD44^−^, CD45^−^ and c-kit^−^9; (ii) BM-isolated adult multilineage inducible cells, characterized as CD29^+^, CD63^+^, Cd81^+^, CD122^+^, CD164^+^, Cd34^−^, Cd36^−^, Cd45^−^ and c-kit^−^10; and (iii) unrestricted somatic stem cells, which express CD34^low^, CD45^−^ and c-kit^low^.^11^ Very small embryonic-like cells are approximately 3–5 μm in diameter, with large nuclei and few cytoplasmic components, and they express CXCR4.^12^,^13^ Unfortunately, the multipotency of these cells has been questioned because of contentious results.^14^,^15^

In addition, scientists have had difficulties maintaining the morphological features and surface marker expression of BM stem cells during their *in vitro* expansion. On culture, BM stem cells change to exhibit mesenchymal-like cellular morphological features and do not maintain the multilineage regenerative function after transplantation *in vivo*. Because of unsuccessful *in vitro* expansion of BM stem cells, only freshly isolated BM cells are used for transplantation.^16^ In addition, although MSC therapy could be used to establish a new clinical paradigm, recent trials have not had encouraging results, largely because of the uncertainty of the immunomodulatory properties of MSC and the low regenerative efficacy of MSC engraftments.^17^–^20^

Recently, the therapeutic efficacy of MSC was suggested to be due to the factors they secreted, or MSC-released exosomes.^21^ The MSC-released exosomes are approximately 0.1–0.5 μm in diameter and contain small RNA and premicroRNAs (premiRNAs).^21^ The MSC-released exosomes have regenerative activity in cardiac cells.^22^ In addition, they exhibit a cellular protective function in hypoxia-induced pulmonary hypertension,^23^ in restoring liver function and in alleviating liver fibrosis.^24^

In addition to MSC-released exosomes, other particles in the circulation have attracted attention. Platelet-rich plasma (PRP) has exhibited regenerative effects.^25^,^26^ Non-nucleated platelets have been studied; however, the regenerative effects of such platelets are believed to be via growth factors released by the platelets.^21^ Although no direct evidence supports the regenerative function of platelets, platelets can facilitate MSC homing^27^ and platelet-released microparticles can promote neural stem cell proliferation and induce neurogenesis.^28^,^29^

Herein we describe a group of non-platelet RNA-containing particles (NPRCP) found in human umbilical cord blood. These particles are approximately 1–5 μm in diameter and most are located in PRP. Electron microscopy revealed that the morphological features of NPRCP differ from those of platelets. In the presence of nucleated cells, NPRCP fuse into non-nucleated cells that further differentiate into octamer-binding transcription factor 4 (OCT4)-expressing stem cells. Herein we describe the origin, characterization and development of NPRCP *in vitro*.

## Methods

### Source of umbilical cord blood

Blood was collected from the umbilical cords of healthy mothers with full-term normal deliveries who had passed the screening test for any known diseases in Beijing Hospital. All mothers had signed consent forms, which was approved by the Beijing Hospital Ethics Committee according to the International Ethical Guidelines for Biomedical Research Involving Human Subjects.^30^ No patient information was provided with the blood samples.

### Antibodies and chemicals

Octamer-binding transcription factor 4 (OCT4), sex-determining region Y 2 (SOX2), DEAD box polypeptide 4 (DDX4) and integrin β1 antibodies were obtained from Santa Cruz Biotechnology (Santa Cruz, CA, USA). Actin and tubulin antibodies were obtained from CWBiotech (Beijing, China). Alexa Fluor goat anti-rabbit IgG was from Invitrogen (Carlsbad, CA, USA). All other chemicals were from Sigma Chemical (St Louis, MO, USA). MicroRNA (miRNA) array hybridization and data analyses were performed by ShanghaiBio Corporation (Shanghai, China).

### Isolation and *in vitro* culture of NPRCP

Umbilical cord blood was stored at 4°C and isolated within 24 h of collection. Whole blood was centrifuged at 200 *g* for 10 min to collect PRP.^31^ The PRP was then centrifuged at 5000 *g* for 10 min. The pellet was resuspended in culture medium (α-minimal essential medium (MEM) containing 20% fetal bovine serum (FBS) and antibiotics) and cultured on collagen-coated plates in a 5% CO_2_ humid incubator at 37°C. The medium was changed every other day. After 2 weeks in culture, the major materials were shiny particles, or NPRCP, which were used for experiments.

### Isolation and culture of particle fusion-derived non-nucleated cells

After removing PRP, blood was incubated in erythrocyte lysis buffer (155 mmol/L NH_4_Cl, 10 mmol/L KHCO_3_ and 0.1 mmol/L EDTA) for 20 min and then centrifuged at 300 *g* for 10 min. The supernatant was transferred to a fresh container and centrifuged at 1000 *g*. After washing with phosphate-buffered saline (PBS), pellets were cultured on collagen-coated plates in α-MEM containing 20% FBS and antibiotics under 5% CO_2_ in a humidity incubator at 37°C. The medium was changed every day for the first 3 days to remove erythrocyte membranes and dead cells and every 2 days thereafter. Particle fusion-derived non-nucleated cells (PFDNC) and precells used for RNA analysis were cultured for 1 week.

### Coculture studies

After at least 2 weeks in culture, NPRCP were transferred to fresh plates at a density of 50 × 10^6^ cells/100 mm plate. Two days later, freshly isolated nucleated cells from umbilical cord blood, with erythrocytes removed, were added to the plates containing NPRCP at a density of 5 × 10^5^ cells/100 mm plate. The medium was changed every other day. Cells were cocultured for at least 3 weeks. Control plates contained only nucleated cells.

### Immunofluorescence staining

Immunofluorescence staining methods were as described previously.^32^ Briefly, NPRCP, PFDNC or prepared cells were washed, fixed in 4% paraformaldehyde for 1 h and then embedded in OCT. Sections were cut and washed with PBS, blocked, incubated in blocking buffer with different antibodies at dilutions ranging from 1 : 50 to 1 : 400 overnight at 4°C, washed, incubated with fluorescent-conjugated secondary antibody for 1 h, washed, counterstained with 4′,6′-diamidino-2-phenylindole (DAPI) and photographed using conventional fluorescence microscopy. Control sections were incubated with non-specific antiserum from the same species as the primary antibody. For Haematoxylin and Eosin (H&E) stain, NPRCP, PFDNC or prepared cells were fixed in 4% paraformaldehyde for 1 h and after washing, dropped onto gelatin-coated slides. Slides were air-dried and then stained for H&E using the protocol found everywhere online.

### Transmission electron microscopy

The embedding methods for electron microscopy were as described previously.^33^ Briefly, enriched NPRCP or PFDNC were fixed in 2% glutaraldehyde and 4% paraformaldehyde in sodium cacodylate buffer, pH 7.3, for 30 min at room temperature. After washing with sodium cacodylate buffer, ice-cold 1% osmium tetroxide in distilled water was added to the cell pellets and samples were shaken gently for 2 h at 4°C. After washing with water, 1% uranyl acetate was added to the cell pellets and samples were left overnight. The cell pellets were then dehydrated with serial ethanol dilution and embedded in Epon at 65°C for 24 h. Ultrathin sections were cut and double stained with uranyl acetate but withnot lead citrate before being examined by electron microscopy.

### Analysis of miRNA

In the present study, RNA was isolated from three groups of NPRCP and three groups of cultured mixed populations containing PFDNC, differentiated PFDNC or precells using the miRNeasy mini kit (Qiagen, Valencia, CA, USA). The quality of the RNA and the ratio of miRNA to small RNA were analysed with an Agilent 2100 Bio-analyser (Agilent Technology, Santa Clara, CA, USA). The ratio of miRNA to small RNA in each sample was calculated automatically by the machine setting, in that the miRNA region was the default setting of 10–40 nucleotides (nt). Microarray hybridization and analysis were performed by ShanghaiBio. The hybridization data were scanned with an Agilent Microarray Scanner, collected using Feature Extraction 10.7 (Agilent Technology) and normalized against that of the internal control signals. Almost 300 of 1300 miRNAs were highly expressed in both groups or only one group. The fold change (*X*) in expression between two groups was analysed using Agilent Gene Spring GX 11.0 with the formula *X* = ((2^A^ + 2^B^ + 2^C^)/3)/((2^X^ + 2^Y^ + 2^Z^)/3), where Groups A, B, C and X, Y, Z represent the normalized individual signals in the two groups. All negative numbers were normalized to zero before calculations were performed. The significance of differences between two groups for the same miRNA were analysed by two-tailed two-sample equal-variance *t*-test. *P* ≤ 0.05 was considered significant (see Tables S1 and S2).

## Results

### Identification of NPRCP in human blood

The circulation contains numerous small particles, including exosomes, cellular membrane vesicles, microparticles and DNA fragments. From our *in vivo* data (W Kong, unpublished data, 2013), we believed that only OCT4-, SOX2- and DDX4-expressing particles were NPRCP. However, purifying NPRCP from a mixed population including platelets and exosomes can be difficult. We believe that centrifugation at 200 *g*, the standard method for PRP isolation, can remove all blood nucleated cells, whereas centrifugation at 5000 *g* can remove most exosomes, because enriching exosomes needs a 0.2 μm filter and 100 000 *g* centrifugation.^22^,^34^ To identify NPRCP and confirm their presence in the extracellular environment, we cultured isolated particles, including platelets, for at least 2 weeks to enrich NPRCP, because the lifespan of platelets is < 10 days. Cultured particles on Days 2 and 13 are shown in Fig. S1a,b, available as Supplementary Material to this paper.

Our data indicate that human umbilical cord blood has a greater variety of particles. After 13 days culture, small shiny particles were the major population in the culture plates. However, we observed many fused particles. To confirm the growth of particles under extracellular conditions, we observed the same field for 5 days and found an increasing number of shiny particles (see Fig. S1 c,d). With longer culture, larger-sized shiny particles showed fission, which may be how they increased in number. The expansion in number occurred only when particles were in a non-eukaryotic cell environment, which suggests that the materials that benefit their expansion are in serum and that nucleated cells may consume the particles. In addition, the number of aggregated shiny particles increased during culture, so the particles may have a tendency to become aggregated.

**Fig. 1:**
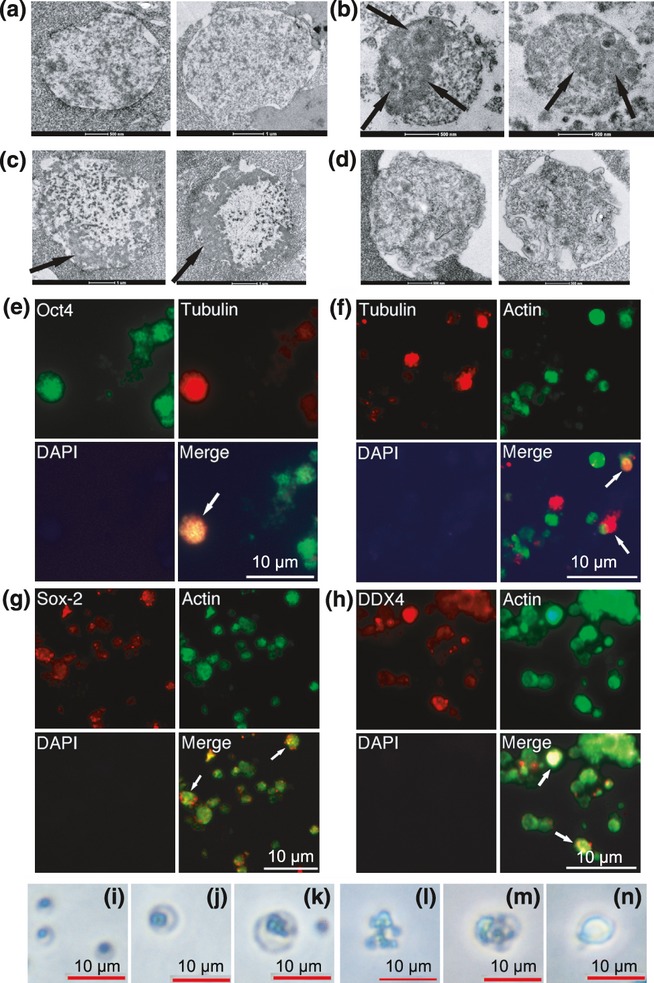
Morphology and expression of non-platelet RNA-containing particles (NPRCP). (a–d) Transmission electron microscopy of four types of NPRCP. One group (a) contains small-sized nuclear granules inside the bilayer membrane. The second group (b) has large dense nuclear bodies (arrows). The third group (c) has more dense nuclear materials. The fourth group (d) has an extremely flexible membrane on the surface and inside of the bodies. (e–h) Culture-enriched NPRCP were stained for coexpression of octamer-binding transcription factor 4 (OCT4) and tubulin (e), tubulin and actin (f), sex-determining region Y 2 (SOX2) and actin (g) and DEAD box polypeptide 4 (DDX4) and actin (h). (i–n) Images of freshly isolated NPRCP. The larger-sized particles are in the fused pattern. Bar in a1 = 500 nm; a2 = 1 um; b = 500 nm; c = 1 um; d = 500 nm.

To confirm that these particles were not platelets with aggregative potential, we examined the detailed structure of these shiny particles by electron microscopy. We observed at least four types of particles, which suggests that the particles are not the same or they change morphologically during differentiation. One group of particles represented small-sized nuclear granules distributed evenly inside the bilayer membrane (Fig. 1a). The second group exhibited one or more large dense nuclear bodies in the centre of small nuclear granules. Each of the dense nuclear bodies had a small core-like structure (Fig. 1b). The third group (Fig. 1c; see also Fig. S1f) possibly differentiated from the second group, with more dense nuclear materials located at one side or in the centre of the bodies. The fourth group (Fig. 1d) had thin membranes on the surface and inside the bodies, which suggests that this type of particle may not have a round shape under normal conditions. This group also contained nuclear granules and fibre-like structures. All these small particles were approximately 1–5 μm in diameter, had a thin bilayer membrane and contained nuclear granules. A small particle next to a shiny particle (see Fig. S1e) suggested the fission hypothesis. Compared with platelets,^35^ our particles did not contain α-granules, cell organelles or glycogen granules, so they were not platelets and therefore were considered non-platelet particles.

We also examined the expression of OCT4, SOX2, DDX4, actin and tubulin in culture-enriched particles. Several OCT4-positive particles were detected (Fig. 1e), but only a relatively larger one coexpressed tubulin (Fig. 1e), which suggests that this particle resulted from fusion of OCT4- and tubulin-positive particles. In addition, some particles expressed both tubulin and actin (Fig. 1f), which suggests fusion. Almost all actin-positive particles expressed SOX2, in a granular pattern (Fig. 1g). Only the relatively larger particles coexpressed DDX4 and actin (Fig. 1h). In addition, OCT4 and SOX2 were costained on same particles (see Fig. S2). None of these particles exhibited DAPI staining. No specific stains were detected in the control sections that were incubated with non-specific antiserum from the same species as the primary antibody (data not shown).

Thus, the particles expressing OCT4, SOX2 and DDX4 and were termed ‘non-platelet RNA-containing particles’ (NPRCP), with strong fusion potential because the growth of the particles may have been by fusion in the culture environment. Images in Fig. 1i–n show the possible stages of growth by fusion of the particles in the culture plates.

### Stem cells release NPRCP

**Fig. 2:**
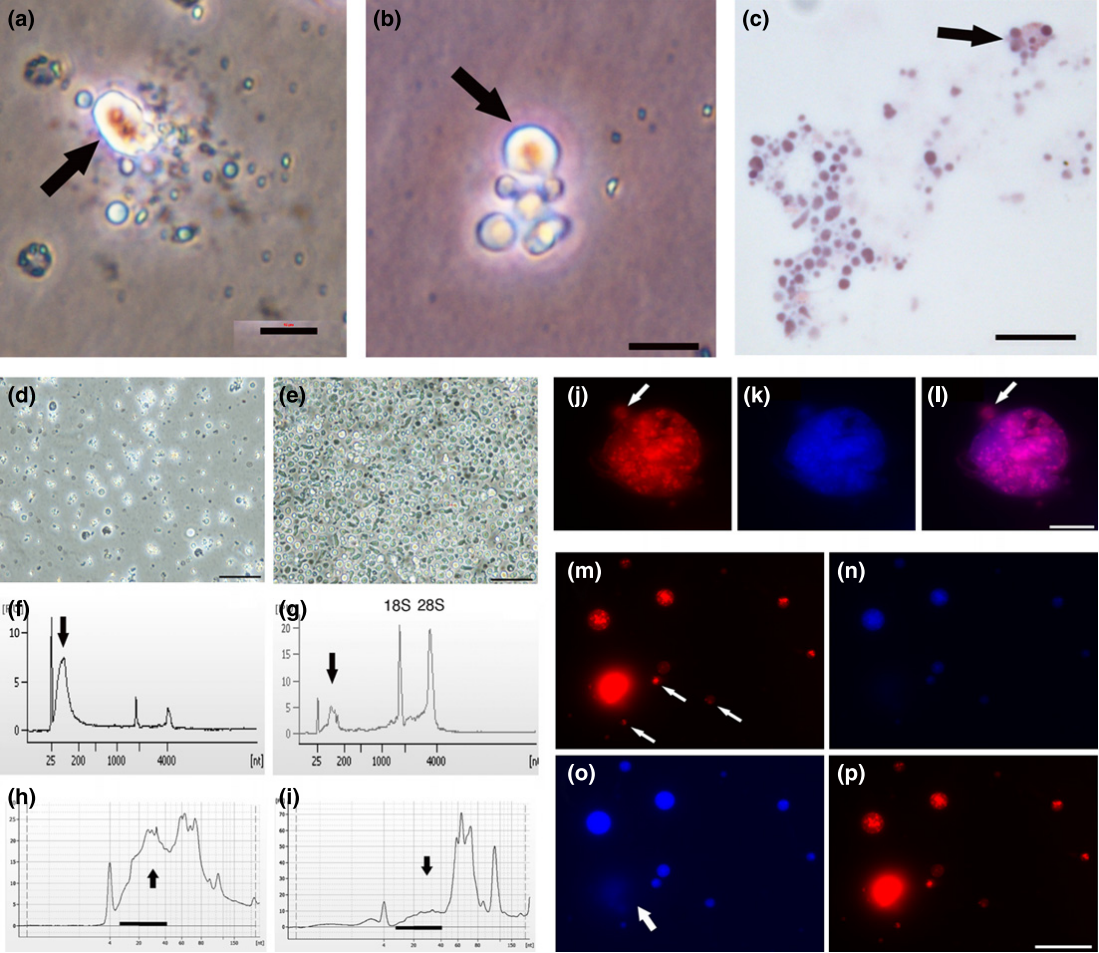
Non-platelet RNA-containing particles (NPRCP) contain small RNA and microRNA (miRNA) and are released from octamer-binding transcription factor 4 (OCT4)-expressing non-nucleated cells following the culture of umbilical cord blood cells. (a,b) Inverted microscopy of cultured NPRCP-releasing cells (arrows). (c) Haematoxylin and eosin staining of NPRCP and their releasing cell (arrow). (d,e) RNA was purified from (d) culture-enriched NPRCP and (e) cultured particle fusion-derived non-nucleated cells (PFDNC) and then analysed. (f,g) The NPRCP (f) contain only small-sized RNA (arrow), whereas mixed PFDNC (g) contain small RNA (arrow) and ribosomal RNAs. The small RNA was further analysed with a micro-gel. (h,i) The ratio of miRNA (10–40 nucleotides; arrow) to small RNA (40–200 nucleotides) was greater for NPRCP (h) than PFDNC (i). (j–p) Immunofluorescence staining for OCT4 and 4′,6′-diamidino-2-phenylindole (DAPI). (l) A non-nucleated cell released one small particle (arrow); (m) another non-nucleated cell released numerous particles (arrows). Bars, 10 μm (a–c,l); 50 μm (d,e); 20 μm (p).

To elucidate the origin of NPRCP, we examined cultured umbilical cord blood cells. Small NPRCP could be observed in the extracellular environment but with a small cellular structure adjacent (Fig. 2a). Numerous small particles were extremely active in the extracellular area, so in earlier observations we mistakenly thought this activation was due to contamination. The action slows when these particles increase in size (Fig. 2b). Thus, NPRCP grow in an extracellular environment and a prestem cell must be present nearby. The presence of a prestem cell strongly suggests that templates of NPRCP are released from prestem cells and are further remodelled or enlarged in the extracellular environment. These released templates may be too small to be observed by standard microscopy. Haematoxylin and eosin staining indicated that numerous small NPRCP locate near a prestem cell (Fig. 2c) that is not stained with haematoxylin, which suggests a non-nucleated prestem cell. The NPRCP exhibited weak haematoxylin staining and little eosin staining, which supports the notion that they contain few nuclear materials and proteins. Thus, NPRCP were released from non-nucleated prestem cells and were enlarged in the extracellular environment. The mechanism of release of NPRCP remains unknown but may be by prestem cell fission.

### Small RNA and miRNA are present in NPRCP

To further identify the genetic materials in the particles, we purified RNA from culture-enriched NPRCP (Fig. 2d). In addition, RNA was isolated from a mixed population of PFDNC (see below) and differentiated precells (Fig. 2e) because of difficulties in separating each type. The NPRCP contained only small RNA, < 250 nt (Fig. 2f), which suggests that the nuclear granules in the particles were *5S* ribosomal RNA or non-coding RNA^36^ and miRNA. In comparison, the mixed population containing differentiated precells contained ribosomal *18S* and *28S* RNA, in addition to a higher ratio of small-sized RNA (Fig. 2g). Furthermore, the contents of the miRNA in the same RNA samples were analysed with a Nano-gel using the 2100 Bioanalyser. The ratio of miRNA to small RNA (71%, 63% and 81% in three NPRCP samples; Fig. 2h) was higher in NPRCP than in mixed PFDNC (33%, 44% and 29% in three PFDNC samples; Fig. 2i). The PFDNC contain more small RNA than miRNA. A miRNA array was performed to compare the expression of miRNA in NPRCP and mixed PFDNC containing differentiated precells. Approximately 300 miRNAs were highly expressed in both groups or only one group (see Tables S1,S2; only miRNAs with significant differences in expression between NPRCP and mixed PFDNC are listed). The high ratio of detectable miRNA in all samples indicates that these small RNAs are not from RNA degradation.

### Prestem cells express OCT4 and do not have a typical nucleus

We examined OCT4 expression in particle-producing prestem cells using immunofluorescence methods. A prestem cell with large vague DAPI-stained granules (Fig. 2k) exhibited OCT4 expression (Fig. 2j) in numerous DAPI granules and a tiny particle located next to the cell (Fig. 2l), which suggests that these particles are being released. These data also support that the nucleus of these prestem cells is not formed. More similar small particle structures, ranging in size from 1 to 6 μm (Fig. 2m), were released from a large prestem cell strongly expressing OCT4 but with almost undetectable DAPI staining (Fig. 2n). Diffuse DAPI staining was detected on this large cellular structure on overexposure of the image (Fig. 2o), which further supports that the prestem cell does not have a nucleus. The two prestem cells in Fig. 2j–l,m–p were larger than the live ones in Fig. 2a,b, possibly because of their flat adhesion on gelatin-coated slides.

### First step of differentiation

**Fig. 3:**
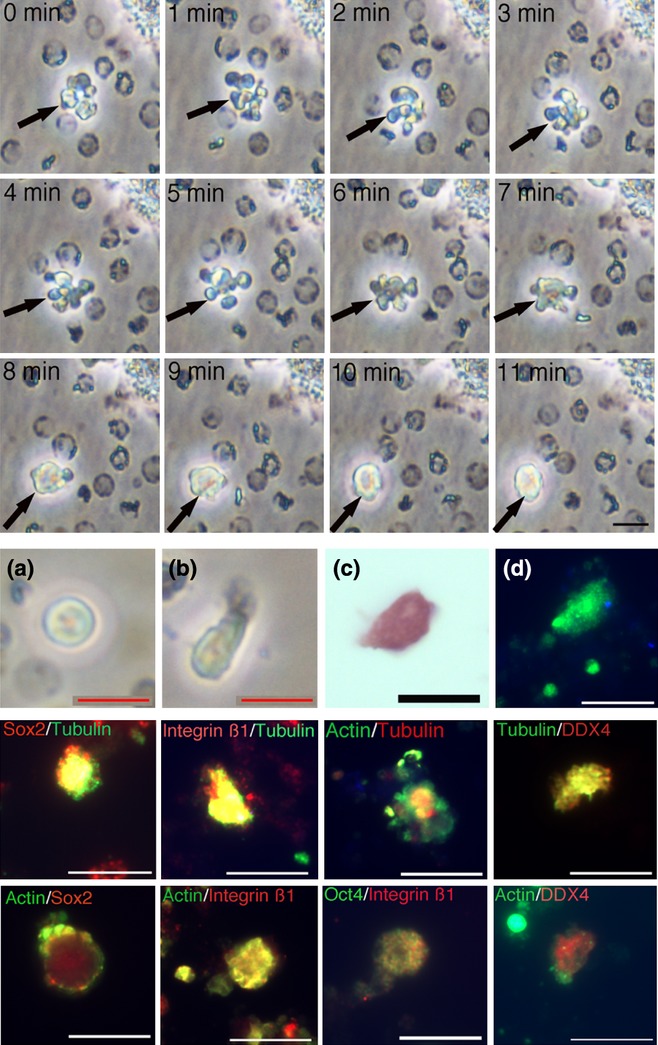
Expression and morphology of particle fusion-derived non-nucleated cells (PFDNC). Time-lapse images show a group of non-platelet RNA-containing particles fusing into a cellular structure in 11 min. Numerous small vesicles surround the structure. (a,b) Cropped images of inactive (a) and active (b) PFDNC. (c) Haematoxylin and eosin and (d) tubulin immunofluorescent staining of PFDNC. The lower two panels show immunofluorescent staining of PFDNC for sex-determining region Y 2 (SOX2), integrin β1, tubulin and DEAD box polypeptide 4 (DDX4). All merged images were stained with 4′,6′-diamidino-2-phenylindole. Images were taken by upright conventional fluorescent microscopy. Bars, 10 μm.

The NPRCP do not form into prestem cells or non-nucleated cells unless in the presence of nucleated cells. Both nucleated cells and NPRCP were observed after coculture for 3 weeks. When nucleated cells were cocultured with NPRCP, they shed a large amount of small cytoplasmic vesicles. The NPRCP possibly fused with cytoplasmic materials released by the nucleated cells to become a group of non-nucleated small cells (Fig. 3). These non-nucleated cells are derived from NPRCP fusion and therefore were called PFDNC. The morphological features of PFDNC have never been described. The PFDNC are approximately 8 μm in diameter and are characterized by their active amoeba-like motion. They can be round when not active (Fig. 3a) but, when active, small PFDNC float in the cultural medium to collect materials by a twisting motion and slow amoeba-like movements (Fig. 3b). Haematoxylin and eosin staining revealed that PFDNC do not have a nucleus (Fig. 3c). Immunofluorescence revealed that PFDNC contain tubulin in their core area (Fig. 3d), which may be the driving force of their amoeba-like motion. Fluorescence costaining revealed that, in addition to expressing tubulin, PFDNC also express integrin β1, SOX2, OCT4, DDX4 and actin (Fig. 3). Actin is expressed on the peripheral membrane of PFDNC, whereas tubulin, SOX2, OCT4 and DDX4 locate in the core area. Integrin β1 locates at both the membrane and core areas (Fig. 3). 4′,6′-Diamidino-2-phenylindole staining did not reveal any nucleus inside the PFDNC, which suggests that they result from NPRCP fusion (see Figs S3,S4). PFDNC were not seen when the nucleated cells were co-present for 3 weeks.

### Repeated entry of PFDNC into nucleated cells

**Fig. 4:**
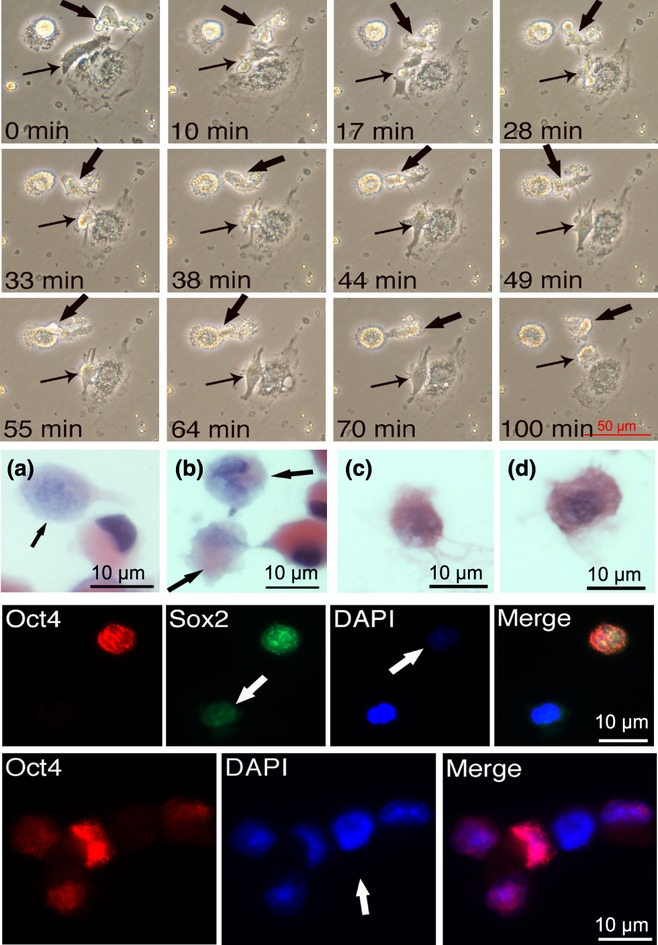
Mesenchymal-like cells are derived from direct differentiation. Time-lapse images of activity of two particle fusion-derived non-nucleated cells (PFDNC; thin and thick arrows) over 100 min. The PFDNC can squeeze their bodies to enter adjacent nucleated cells (thin arrow at 28 min and thick arrow at 55 min). (a–d) Haematoxylin and eosin staining of differentiated PFDNC. Haematoxylin staining is uneven and weak (a,b; arrows). Eosin staining is dense in large-sized precells (c,d; arrows). The upper panels of immunoflurescent images show immunofluorescence staining for octamer-binding transcription factor 4 (OCT4), sex-determining region Y 2 (SOX2) and 4′,6′-diamidino-2-phenylindole (DAPI) in two cells; OCT4 is expressed only with weak DAPI staining in PFDNC (arrow in DAPI) and not in strong DAPI-stained cells. In comparison, SOX2 is expressed stronger in weak DAPI-stained PFDNC and weaker in strong DAPI-stained cells. Images in the lower panel show that OCT4 is expressed only in weak, and not in strong, DAPI-stained cells.

Because of their amoeba-like motion, PFDNC could squeeze their bodies to enter nucleated cells. The PFDNC entered the nuclear or cytoplasmic areas of nucleated mesenchymal-like cells. Time-lapse images shown in Fig. 4 show two PFDNC twisting their bodies to approach the nucleated cells. The PFDNC entered adjacent nucleated cells, which suggests that they were collecting materials from nucleated cells for their development. The PFDNC could spend 10 min or longer inside the nucleated cells or could repeatedly enter one or more cells. Similar activities have been described for neutrophils and monocytes, which can pass through endothelial cells or fibroblasts.^37^,^38^ However, we do not believe that PFDNC are monocytes or neutrophils because PFDNC do not have a nucleus. In our *in vivo* studies, all extravasated materials were transplanted NPRCP, which indicates that the specific migration of PFDNC is by actively passing though blood vessels (W Kong, unpublished data, 2013). After exiting nucleated cells, PFDNC continued to collect small particles in the environment (video records are available on request to the authors) and became differentiated PFDNC that could be 10 μm or larger and exhibited a slow crawling motion after exiting cells. Although PFDNC are possibly the result of fusion of NPRCP, they appeared only when eukaryotic cells were present, which suggests that materials from the nucleated cells participate in the formation of PFDNC. Electron microscopy confirmed that PFDNC are non-nucleated cells created by one NPRCP engulfed by a small amount of cellular material. Two precellular structures of approximately 5 and 8 μm, with an NPRCP in the centre (see Fig. S5), suggest that the core of PFDNC derives from NPRCP. The peripheral areas of these structures are filled with numerous tiny particles, which further supports the active ‘catching’ characteristic of PFDNC.

### Differentiation into mesenchymal-like cells

When PFDNC increased in size by integrating collected particles from the environment and nucleated cells, or when they became differentiated, they stopped entering nucleated cells, possibly because of difficulties in squeezing their large bodies into nucleated cells or because they had collected what they needed for further transformation. Haematoxylin and eosin staining revealed that differentiated PFDNC did not have a normal nucleus compared with adjacent nucleated cells. Haematoxylin and eosin weakly stained all areas of differentiated PFDNC (Fig. 4a,b). In comparison, eosin staining was denser in large-sized differentiated PFDNC or precells (Fig. 4c,d), so differentiated PFDNC represent the combination of large-sized NPRCP and cytoplasmic materials. Furthermore, OCT4 (Fig. 4) was expressed all over weak DAPI-stained differentiated PFDNC, but not strong DAPI-stained cells, which, in comparison, still exhibited SOX2 staining. These data indicate that the transient expression of OCT4 is shorter than that of SOX2 on these PFDNC-derived cells. In addition, the denser the DAPI stain, the weaker the OCT4 stain (see Fig. 4), indicating that PFDNC-derived nucleated cells lose OCT4 expression when they became fully differentiated. Thus, OCT4 expression in differentiated PFDNC is transient. The differentiated PFDNC undergo cellularization to become mesenchymal-like cells.

### Fusion differentiation to large cells

**Fig. 5:**
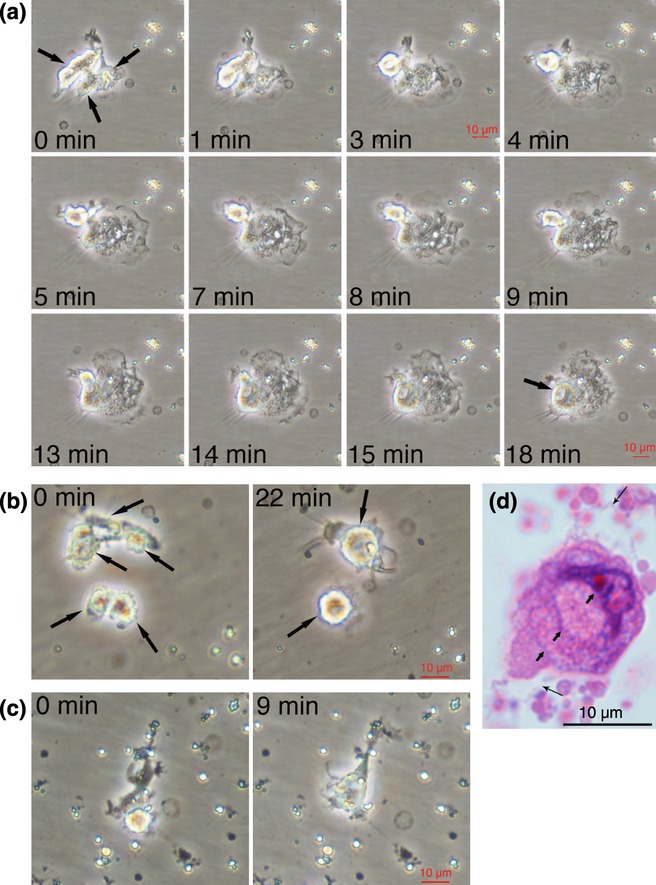
Particle fusion-derived non-nucleated cells (PFDNC) fuse into mesenchymal-like cells. (a) Time-lapse images show fusion of subcellular structure. Three subcellular structures (arrows) fuse to become a cell within 18 min. (b) Two pairs of differentiated PFDNC fuse to each other to become two precells. (c) A precell fuses to a cytoplasmic membrane to become a eukaryotic cell in 9 min. Images were taken by inverted microscopy. (d) Haematoxylin and eosin staining of a cell with multiple layers inside (thick arrows) the thin, loose outer membrane (thin arrows).

Although mature PFDNC can directly transdifferentiate into nucleated mesenchymal-like cells, we also found that differentiated PFDNC fused to each other to further increase their size and become large cells. Time-lapse images (Fig. 5a) show three PFDNC, or subcellular structures, that fused together or were engulfed by each other to become one cellular structure in 18 min. Two pairs of differentiated PFDNC (Fig. 5b) fused to each other to become two precells that were round and without a typical cellular membrane (videos are available on request from the authors). The fusion between two PFDNC was not a simple membrane connection. Instead, a whole PFDNC appeared to be engulfed by another one. This process could occur within a few minutes. Having a loose membrane may facilitate this process. A precell entered a cytoplasmic membrane to become a mesenchymal-like cell within a few minutes (Fig. 5c). Haematoxylin and eosin staining (Fig. 5d) showed that, in a newly formed cell with a loose cellular membrane, three circular-arranged layers from the centre to the outer membrane were packed together. Each layer had a clear boundary, so this cell may have derived from engulfing three subcellular structures. Haematoxylin staining was in a circular shape, so this cell was still undergoing nuclear programming. The nuclei of the new cells may be programmed by the contents of NPRCP and the collected genetic materials, whereas the outer layers may be derived from the cytoplasmic portions of other nucleated cells.

### Fusion differentiation to stem cells

**Fig. 6:**
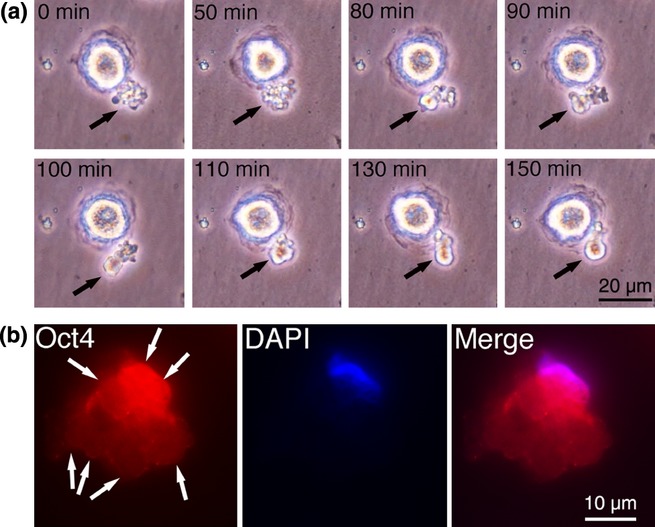
Non-platelet RNA-containing particles (NPRCP) fusion differentiation. Cocultured NPRCP were examined by microscopy and video recorded. (a) Time-lapse images were cropped from snapshots of a video recording to show a group of small particles fusing into a cellular structure in 150 min. (b) Staining for octamer-binding transcription factor 4 (OCT4) expression in a fusion-derived cell.

We found that NPRCP could fuse into larger cellular structures. Small particles could aggregate, fuse and transform into a cellular structure after more than 2 h (Fig. 6a). This fusion process could also occur in a few minutes, which may be due to fewer participating NPRCP (Fig. 3). This final cellular product did not exhibit a standard cytoplasmic membrane and therefore needed to stay close to or partially inside the cellular membrane of the adjacent nucleated cell, which suggests that its further development depended on this nucleated cell. The further developmental procedure may include obtaining genetic materials from the adjacent cells for nuclear programming and cell-type lineage determination. A cellular structure showed multiple small OCT4-expressing vesicles (Fig. 6b), which may be from the NPRCP. 4′,6′-Diamidino-2-phenylindole staining was located at the edge of this aggregate, which suggests that the nuclear materials are obtained during the aggregation for nuclear programming.

### NPRCP-fusion-derived large cells are stem cells

**Fig. 7:**
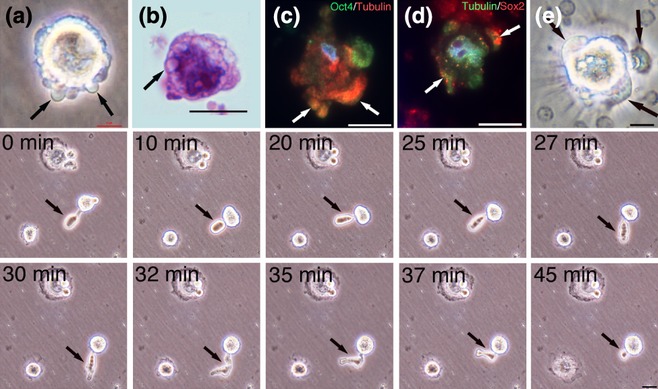
Fusion-derived large cells release small non-nucleated cells. (a) A live cell in the culture plate protrudes at least two cellular portions (arrows). (b) Haematoxylin and eosin staining of a large cell shows irregular haematoxylin staining. The protruding cytoplasmic portions show slight haematoxylin staining at the outer edges (arrow). (c,d) Immunofluorescence staining of protruding portions shows a large amount of tubulin, octamer-binding transcription factor 4 (OCT4) and sex-determining region Y 2 (SOX2) expression (arrows). (e) A live cell releases a small non-nucleated cell and shows two more protrusions (arrows). The lower panels show time-lapse images of a cellular protrusion (arrows) with extremely active motion. Bars, 10 μm (a–e); 20 μm (lower panels).

We found that one group of large cells was extremely active. They constantly showed protrusion of one or more subcellular portions from their bodies. A live cell in the culture plate showed protrusion of at least two subcellular portions (Fig. 7a). The protrusions had a smooth surface and protruded from different sides of the cell. Time-lapse studies (Fig. 7) showed an extremely active cellular protrusion. The protruded portion can bud off from the large cells (data not shown). Haematoxylin and eosin staining revealed a large cell with an irregular haematoxylin-positive nucleus (Fig. 7b). The protruding cytoplasmic portions showed slight haematoxylin staining at the outer edges (Fig. 7b). No typical nucleus was observed in this cell. We believe that tubulin may be the driving force for their active motion. Immunofluorescence staining further supported that the budding protrusions contained a large amount of tubulin and expressed OCT4 and SOX2 (Fig. 7c,d). However, no DAPI staining was detected in these protrusions, which suggests that the protrusions are non-nucleated prestem cells. The morphological features of the tubulin-expressing protrusions were similar to those of PFDNC, so they may be subcellular structures of the cells that were differentiated after multiple NPRCP fusion. Figure S6 shows individual images of the merged figures in Fig. 7c,d. The protrusions were non-nucleated small cells that were released from the large stem cell (Fig. 7e). The cytoplasmic portion-protruding stem cells may be derived from the multiple NPRCP fusion-derived stem cells (see Fig. 6) that are differentiated after fusion and incubation, because these protruded cells did not have a nucleus.

## Discussion

Herein we describe NPRCP from human umbilical cord blood that express OCT4, SOX2 and DDX4 and contain miRNAs and small RNAs. When nucleated cells were present, NPRCP transformed into non-nucleated small active cells, namely PFDNC, that further differentiated into mesenchymal-like cells. The differentiation of PFDNC may involve a mechanism whereby DNA fragments are collected from nucleated or damaged cells. In addition, multiple NPRCP could fuse into large cellular structures that are prestem cells because their nucleus is not fully formed. These large cellular structures expressed OCT4 and SOX2 and released small non-nucleated cells that are similar to PFDNC. Our data provide strong evidence that NPRCP and PFDNC are precellular structures that exist in human blood.

The NPRCP are released by small stem cells that strongly express OCT4 but are weakly stained with DAPI. In extracellular environments, NPRCP can grow, possibly by collecting the circulation molecules. Further, we have found that NPRCP can fuse into PFDNC. The appearance of PFDNC occurs only when eukaryotic cells are present with NPRCP, which indicates that the membrane or cytoplasmic proteins from eukaryotic cells are essential for NPRCP to fuse into PFDNC. In addition, multiple NPRCP could fuse into large cellular structures that further differentiate into large stem cells and release small non-nucleated stem cells or PFDNC. Although we had many difficulties isolating NPRCP from various differentiating stages, from our electron microscopy studies we believe that NPRCP are not identical, so more investigations are needed.

Stem cell self-renewal was previously believed to be by asymmetric mitotic division.^1^ Our results indicate that a group of small non-nucleated stem cells, or prestem cells, can release hundreds of NPRCP that further grow, fuse and assemble into nucleated cells. A single prestem cell can produce many NPRCP, possibly by fission. Both NPRCP and the small non-nucleated stem cells contain materials similar to germinal materials in eggs. The mechanism of producing NPRCP may involve fission of the germinal materials and not mitosis that involves DNA or chromosomes. In trace amount DNA-induced nuclear programming, described more than 20 years ago, 5 ng phage DNA could form into a new nucleus after injection into *Xenopus* eggs.^39^ The assembly of the mini nuclei does not depend on a specific DNA sequence and does not require the presence of the egg nucleus, centromere or telomere.^39^ That report indicates that *de novo* nuclear programming can occur as a result of the presence of a small amount of DNA and that the egg materials are essential for nuclear formation. Other reports also indicate that material in germinal vesicles is essential for all successful nuclear cloning.^40^

The nuclear components were in an evenly distributed pattern or as large, dense granules in NPRCP, which is similar to that described in primordial germ cells in lower species.^41^ In addition, similar to chromatoid bodies (the germ line-specific RNA granules that express DDX4 (synonym: VASA), NPRCP expressed DDX4 and contained small RNA, supporting the notion that NPRCP may have similar components to that of primordial germ cells or chromatoid bodies. Our electron microscopy, immunofluorescence and RNA analyses of components suggest that the content of RNA proteins in NPRCP is similar to that in germinal materials. These materials undergo fission to form more NPRCP and also undergo fusion to form PFDNC. Our data strongly support that the mechanism of the differentiation of the PFDNC is by *de novo* nuclear programming in the RNA-containing materials. In addition, our data do not support that the release of PFDNC by the large stem cells is via any kind mitotic cellular division. The non-nucleated cells are not from mitotic asymmetric division. Future investigations are needed to elucidate the mechanism of by which PFDNC are produced.

We report on two types of stem cells derived from NPRCP. One is from direct differentiation of PFDNC that lose OCT4 expression when they become differentiated mesenchymal-like cells. Another is the large cells that protrude large cytoplasmic portions or release small non-nucleated cells. Although protruding and budding off the non-nucleated cells seems an asymmetric procedure, no mitotic division is involved in this procedure. We believe that this asymmetric budding is possibly driven by tubulin. In our *in vivo* studies (W Kong, unpublished data, 2013) we have found that these large stem cells can help regenerate larger tissues, such as renal tubules. They could quickly produce a large amount of small cells if needed. They also participate in the differentiation and became tissue-specific cells. Our data indicate that these non-nucleated cells are prestem cells or true stem cells that release NPRCP and do not have a nucleus. These stem cells may have only stem cell nuclear materials and not tissue-specific genetic cellular information, so their multipotency may be acquired from adjacent nucleated cells during non-nucleated small cell differentiation. The NPRCP-releasing prestem cells can be consumed or fully differentiate to nucleated cells, depending on the microenvironment; however, some NPRCP can grow larger to become new prestem cells to release new NPRCP. Thus, these prestem cells are not long-lived, but are recycled.

The identification of NPRCP and PFDNC provides new potential for cell therapy. Because they do not have a nucleus, NPRCP and PFDNC may not induce rejection, can regenerate into tissue-specific stem cells after transplantation and could be good candidates for use in cell therapy.

## SUPPORTING INFORMATION

**Figure S1**. Non-platelet RNA-containing particles (NPRCP) can be expanded in culture. Images show the cultured particles on (a) Days 2 and (b) 13. Change in NPRCP number in the same area before (c) and after 5 days (d) in culture. The NPRCP had been cultured for 20 days before examined for electron microscopy. (e,f) Electron microscopy of culture-enriched NPRCP. Bars, 50 μm (a–d); 200 nm (e); 500 nm (f).

**Figure S2**. Coexpression of OCT4 and SOX2 in non-platelet RNA-containing particles (NPRCP). The NPRCP were cultured on collagen-coated cover slides for 2 weeks, then fixed for immunofluorescence staining. OCT4 and SOX2 were coexpressed in a granule pattern on NPRCP (arrows). Bar, 10 μm.

**Figure S3**. Individual images for merged fluorescent images of particle fusion-derived non-nucleated cells (PFDNC) in the upper bottom panel of Fig. 3. Except for a trace amount of nuclear materials, 4′,6′-diamidino-2-phenylindole did not stain any nuclear structures. Bar, 10 μm.

**Figure S4**. Individual images for merged fluorescent images of particle fusion-derived non-nucleated cells (PFDNC) in the bottom panel of Fig. 3. Except for a trace amount of nuclear materials, 4′,6′-diamidino-2-phenylindole did not stain any nuclear structures. Bars, 10 μm.

**Figure S5**. Particle fusion-derived non-nucleated cells (PFDNC) are derived from a collection of cytoplasmic material by non-platelet RNA-containing particles (NPRCP). Electron microscopy of two precellular structures, approximately 5 μm (a) and 8 μm (b) with an NPRCP in the centre (arrows), which suggests that the core of the PFDNC is derived from NPRCP.

**Figure S6**. Individual images (a,b) of merged images of large cytoplasmic portion-protruding cells in Fig. 7c,d, respectively. The arrow points to structures with morphological features of particle fusion-derived non-nucleated cells (PFDNC). Bars, 10 μm.

**Table 1(S1)**. Overexpressed microRNA in non-platelet RNA-containing particles (NPRCP) compared with precells and particle fusion-derived non-nucleated cells (PFDNC).

**Table 2(S2)**. MicroRNAs with decreased expression in non-platelet RNA-containing particles (NPRCP) compared with precells and particle fusion-derived non-nucleated cells (PFDNC).
