# Supplementary material for: Pre-stem cell formation by non-platelet RNA-containing particle fusion
Source: Clin Exp Pharmacol Physiol. 2013 Jun 21;40(7):412–21. doi: 10.1111/1440-1681.12101 (PMC3748798; doi:10.1111/1440-1681.12101)
Supplement: Supplementary file 1 [file cep0040-0412-SD1.pdf]

# Supplementary Figures and Tables

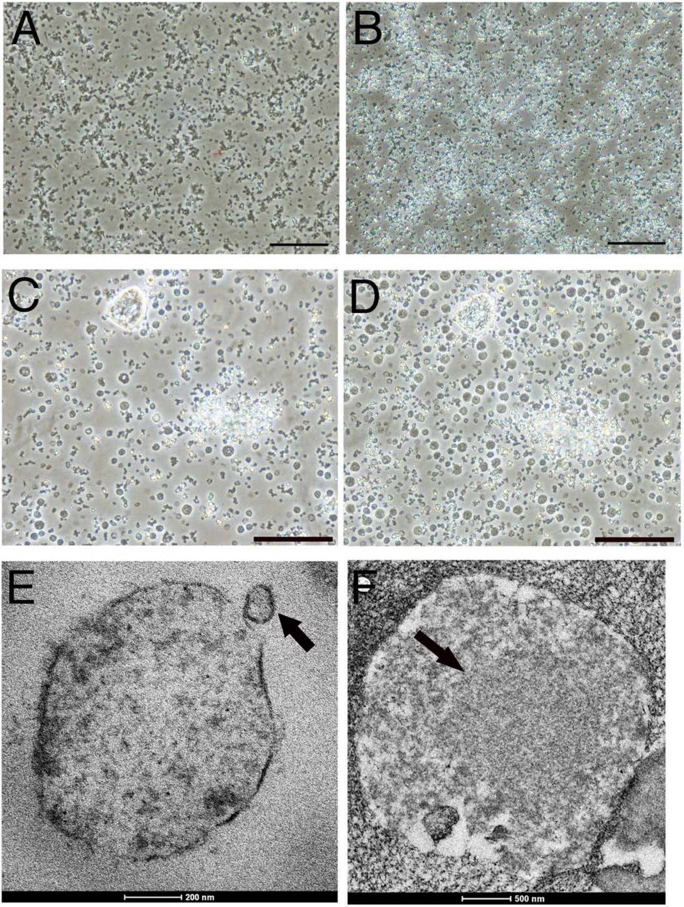


Supplementary Figure 1. NPRCPs can be expanded in culture. Images show the cultured particles on days 2 (A) and 13 (B). Change in NPRCP number in the same area at day 5 of cultivation (C, D). NPRCPs had been cultured for 20 days before this experiment. Electron microscopy of culture-enriched NPRCPs (E and F, arrows). Scale bars in A-D = 50 μm; E = 200 nm; F = 500 nm.

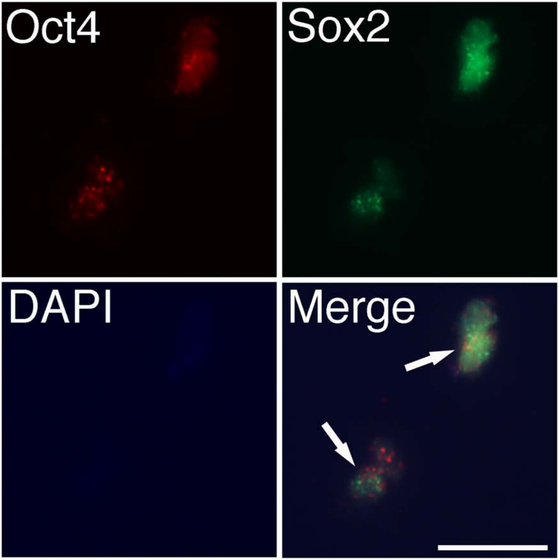


Supplementary Figure 2. Co-expression of Oct4 and Sox2 in NPRCPs. NPRCPs were cultured on collagen-coated cover slides for 2 weeks, then fixed for immunofluorescence staining. Oct4 and Sox2 were co-expressed in a granule pattern on NPRCPs (arrows). Scale bar = 10 μm.

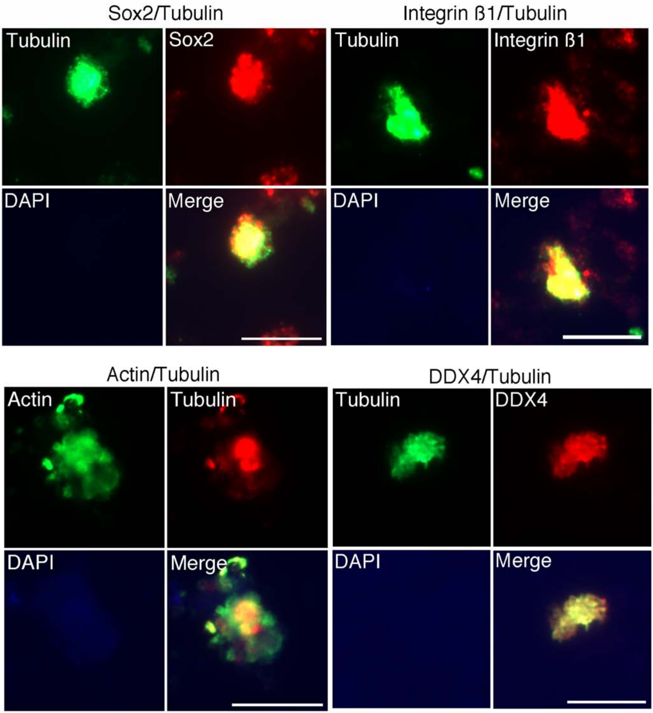


Supplementary Figure 3. Individual images for merged fluorescent images of PFDNCs in the upper bottom paneal of Figure 3. Except for a trace amount of nuclear materials, DAPI did not stain any nuclear structures.
Scale bars = 10 μm.

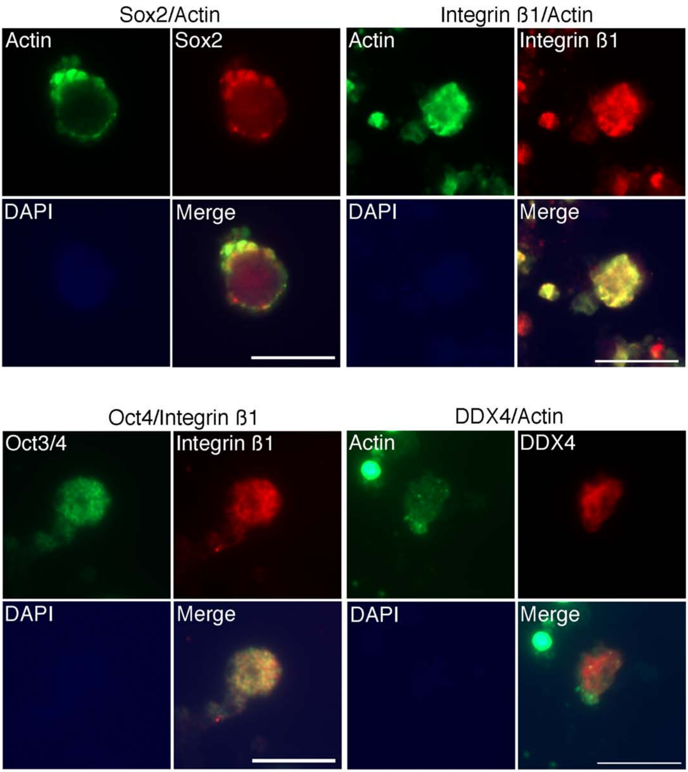


Supplementary Figure 4. Individual images for merged fluorescent images of PFDNCs in the bottom panel of Figure 3. Except for a trace amount of nuclear materials, DAPI did not stain any nuclear structures. Scale bars = 10 μm.

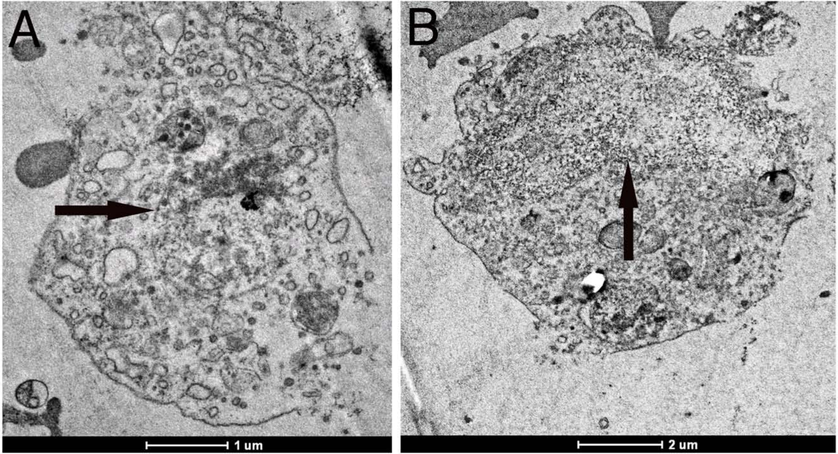


Supplementary Figure 5. PFDNCs are derived from a collection of cytoplasmic material by NPRCPs. Electron microscopy of 2 pre-celular structures, each about 5 µm (A) and 8 µm (B) with an NPRCP in the center (arrows), which suggests that the core of PFDNCs are derived from NPRCPs.

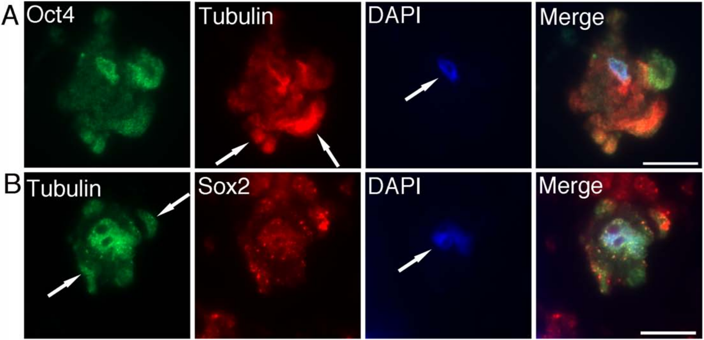


Supplementary Figure 6. Individual images (A and B) of merged images of large cytoplasmic portion-protruding cells in Figure 7C and 7D, respectively. The arrow points to structures with PFDNC morphologic features. Scale bars = 10 μm.

Table 1. Overexpressed miRNA in NPRCPs compared to pre-cells and PFDNCs

| Systematic_name | Active_sequence | Fold Change | p value |
|---|---|---|---|
| hsa-miR-1182 | GTCACATCCCTCCCA | 42.91 | 0.00 |
| hsa-miR-1183 | TGCCCACTCTCACCA | 26.46 | 0.02 |
| hsa-miR-1224-5p | CCACCTCCCGA | 6.45 | 0.02 |
| hsa-miR-1225-5p | CCCCCCACTGG | 3.74 | 0.03 |
| hsa-miR-1275 | GACAGCCTCTCCCC | 6.92 | 0.00 |
| hsa-miR-134 | CCCCTCTGGTCAA | 3.43 | 0.03 |
| hsa-miR-150* | CTGTCCCCCAGGC | 18.87 | 0.03 |
| hsa-miR-1973 | TATGCTACCTTTGCACG | 10.42 | 0.02 |
| hsa-miR-2861 | CCGCCCACCGC | 6.66 | 0.00 |
| hsa-miR-30c-1* | GGAGTAAACAACCCTCTCC | 7.66 | 0.02 |
| hsa-miR-3138 | ACTCCCTCTACCTCACT | 3.88 | 0.02 |
| hsa-miR-3141 | TCCTCCTCCACCCG | 7.74 | 0.02 |
| hsa-miR-3154 | TCTGCTCCCAACTCC | 42.27 | 0.00 |
| hsa-miR-3162 | CTCCCCACCCTTCT | 3.72 | 0.04 |
| hsa-miR-3196 | GAGGCCCCTGCCG | 4.49 | 0.04 |
| hsa-miR-3198 | TCTCCATTCCCCAGG | 1.46 | 0.05 |
| hsa-miR-3202 | ATTAAAGCTCTTCTCCCTT | 14.14 | 0.02 |
| hsa-miR-320c | ACCCTCTCAACCCAG | 7.63 | 0.00 |
| hsa-miR-345 | GAGCCCTGGACTAG | 5.25 | 0.04 |
| hsa-miR-3648 | CCCTCGGCGATCC | 4.65 | 0.04 |
| hsa-miR-3652 | TCCTCACACCTCCAGC | 3.99 | 0.00 |
| hsa-miR-3656 | CCACCCCCGCAC | 5.85 | 0.03 |
| hsa-miR-3663-3p | GCGCCCGGCCT | 5.36 | 0.03 |
| hsa-miR-3665 | CGCCGCCCCG | 3.46 | 0.05 |
| hsa-miR-3667-5p | ACCTTCTCCTCAATGGG | 6.18 | 0.02 |
| hsa-miR-3679-5p | TCCCCTTCCCTGCC | 10.00 | 0.00 |
| hsa-miR-3682 | CTACCTCCACCTGTATC | 22.14 | 0.03 |
| hsa-miR-3713 | ACCATCCCCAAACGG | 17.06 | 0.00 |
| hsa-miR-3917 | CCCACCTGCTCAGT | 65.19 | 0.00 |
| hsa-miR-422a | GCCTTCTGACCCTA | 68.13 | 0.00 |
| hsa-miR-4253 | ACCCCCTGGACATGC | 13.41 | 0.03 |
| hsa-miR-4257 | CTCAGTCCCCACCT | 7.23 | 0.05 |
| hsa-miR-4271 | CCCCACCTTTTCTTCC | 6.45 | 0.03 |
| hsa-miR-4281 | CCCCCCTCCCCG | 7.85 | 0.03 |
| hsa-miR-4298 | CTGCCTCCTCCTCC | 11.24 | 0.02 |
| hsa-miR-4314 | CTGTCCCATTTCCCA | 64.19 | 0.00 |
| hsa-miR-4327 | CCAGTCCCCCATGC | 17.76 | 0.04 |
| hsa-miR-451 | AACTCAGTAATGGTAACGGTTT | 3.77 | 0.01 |
| hsa-miR-483-5p | CTCCCTTCTTTCCTC | 4.76 | 0.01 |
| hsa-miR-498 | GAAAAACGCCCCCTGGC | 64.61 | 0.01 |
| hsa-miR-514b-5p | ATGATTGCCTCCCTCTT | 76.29 | 0.00 |
| hsa-miR-526b | ACAGAAAGTGCTTCCCTC | 28.75 | 0.00 |
| hsa-miR-572 | TGGGCCACCGCCG | 3.62 | 0.01 |
| hsa-miR-575 | GCTCCTGTCCAACTGGCT | 5.59 | 0.01 |
| hsa-miR-638 | AGGCCGCCACCCGC | 5.46 | 0.00 |
| hsa-miR-642b | GGGTCCCTCTCCAA | 5.20 | 0.01 |
| hsa-miR-762 | GCTCGGCCCCGG | 13.75 | 0.05 |

| | | | |
|---|---|---|---|
| hsa-miR-765 | CATCACCTTCCTTCTCCT | 4.67 | 0.01 |
| hsa-miR-769-3p | AACCAAGACCCCGGAG | 15.75 | 0.02 |
| hsa-miR-877 | CCCTGCGCCATCT | 98.91 | 0.01 |
| hsa-miR-936 | CTGCGATTCCTCCCT | 29.97 | 0.00 |

Note: almost 300 of 1300 microRNAs were highly expressed in both groups or only one group. Only the miRNAs that were significantly higher in level in NPRCPs than the mixed PFDNCs are listed.

Table 2. MicroRNAs with decreased expression in NPRCPs as compared to pre-cells and PFDNCs.

| Systematic_name | Active_sequence | Fold change | p value |
|---|---|---|---|
| hsa-let-7a | AACTATACAACCTACTACCT | 0.178 | 0.04 |
| hsa-let-7g | AACTGTACAAACTACTACCTC | 0.145 | 0.02 |
| hsa-miR-101 | TTCAGTTATCACAGTACTGT | 0.369 | 0.02 |
| hsa-miR-10a | CACAAATTCGGATCTACAGGG | 0.018 | 0.00 |
| hsa-miR-125b | TCACAAGTTAGGGTCTC | 0.018 | 0.00 |
| hsa-miR-1260 | TGGTGGCAGAGGTGG | 0.261 | 0.05 |
| hsa-miR-128 | AAAGAGACCGGTTCACTGT | 0.416 | 0.05 |
| hsa-miR-1280 | GGGTGGCAGCGG | 0.764 | 0.02 |
| hsa-miR-140-3p | CCGTGGTTCTACCCT | 0.264 | 0.03 |
| hsa-miR-140-5p | CTACCATAGGGTAAAACCACT | 0.261 | 0.03 |
| hsa-miR-142-3p | TCCATAAAGTAGGAAACACTACA | 0.112 | 0.00 |
| hsa-miR-142-5p | AGTAGTGCTTTCTACTTTA | 0.100 | 0.00 |
| hsa-miR-148a | ACAAAGTTCTGTAGTGCACT | 0.292 | 0.04 |
| hsa-miR-150 | CACTGGTACAAGGGTTGG | 0.024 | 0.00 |
| hsa-miR-155 | ACCCCTATCACGATTAG | 0.155 | 0.00 |
| hsa-miR-15a | CACAAACCATTATGTGCTGCT | 0.466 | 0.05 |
| hsa-miR-16-2* | TAAAGCAGCACAGTAATATTGG | 0.035 | 0.00 |
| hsa-miR-181a | ACTCACCGACAGCGT | 0.120 | 0.00 |
| hsa-miR-181a* | GGTACAATCAACGGTCGA | 0.021 | 0.00 |
| hsa-miR-181c | ACTCACCGACAGGTTGAAT | 0.017 | 0.00 |
| hsa-miR-181d | ACCCACCGACAACAATG | 0.051 | 0.00 |
| hsa-miR-186 | AGCCCAAAAGGAGAATTCTTT | 0.300 | 0.02 |
| hsa-miR-196b | CCCAACAACAGGAAACTACC | 0.042 | 0.03 |
| hsa-miR-20a* | CTTTAAGTGCTCATAATGCAG | 0.031 | 0.00 |
| hsa-miR-21* | ACAGCCCATCGACTG | 0.033 | 0.00 |
| hsa-miR-222 | ACCCAGTAGCCAG | 0.028 | 0.00 |
| hsa-miR-223* | AACTCAGCTTGTCAAATACACG | 0.051 | 0.03 |
| hsa-miR-23b | GGTAATCCCTGGCAATG | 0.358 | 0.05 |
| hsa-miR-26a | AGCCTATCCTGGATT | 0.259 | 0.02 |
| hsa-miR-26b | ACCTATCCTGAATTACTTGA | 0.226 | 0.04 |
| hsa-miR-29a | TAACCGATTTCAGATGGTGC | 0.233 | 0.02 |
| hsa-miR-29b | AACACTGATTTCAAATGGTGC | 0.165 | 0.03 |
| hsa-miR-29c | TAACCGATTTCAAATGGTGCTA | 0.245 | 0.01 |
| hsa-miR-30b | AGCTGAGTGTAGGATGTT | 0.189 | 0.03 |
| hsa-miR-30c | GCTGAGAGTGTAGGATGT | 0.203 | 0.04 |
| hsa-miR-30e | CTTCCAGTCAAGGATGT | 0.355 | 0.01 |
| hsa-miR-324-3p | CCAGCAGCACCTGGGG | 0.267 | 0.03 |
| hsa-miR-338-3p | CAACAAAATCACTGATGCTGG | 0.204 | 0.02 |
| hsa-miR-33a | TGCAATGCAACTACAATGCAC | 0.034 | 0.00 |
| hsa-miR-342-3p | ACGGGTGCGATTTCTG | 0.132 | 0.01 |
| hsa-miR-342-5p | TCAATCACAGATAGCACCC | 0.006 | 0.00 |
| hsa-miR-361-3p | AAATCAGAATCACACCTGGG | 0.013 | 0.00 |
| hsa-miR-362-3p | TGAATCCTTGAATAGGTGTG | 0.026 | 0.00 |
| hsa-miR-362-5p | ACTCACACCTAGGTTCC | 0.024 | 0.00 |
| hsa-miR-363 | TACAGATGGATACCGTGCA | 0.249 | 0.03 |

| | | | |
|---|---|---|---|
| hsa-miR-374a | CACTTATCAGGTTGTATTATAA | 0.243 | 0.05 |
| hsa-miR-424 | TTCAAAACATGAATTGCTGCTG | 0.159 | 0.01 |
| hsa-miR-4291 | AGCTGTTCCTGCTGAA | 0.055 | 0.00 |
| hsa-miR-4299 | GCCTCTCATGTCACC | 0.184 | 0.03 |
| hsa-miR-4317 | AAACTCCCTGGCAATG | 0.062 | 0.00 |
| hsa-miR-493* | AATGAAAGCCTACCATGTAC | 0.088 | 0.02 |
| hsa-miR-500a* | CAGAATCCTTGCCCAGGT | 0.084 | 0.02 |
| hsa-miR-505 | AGGAAACCAGCAAGTGTTG | 0.047 | 0.00 |
| hsa-miR-532-3p | TGCAAGCCTTGGGTG | 0.051 | 0.00 |
| hsa-miR-551b | CTGAAACCAAGTATGGGTCGC | 0.024 | 0.00 |
| hsa-miR-590-5p | CTGCACTTTTATGAATAAGCTC | 0.279 | 0.01 |
| hsa-miR-660 | CAACTCCGATATGCAATGG | 0.295 | 0.02 |
| hsa-miR-99a | CACAAGATCGGATCTACGG | 0.025 | 0.00 |

Note: almost 300 of 1300 microRNAs were highly expressed in both groups or only one group. Only the miRNAs that were significantly lower in level in NPRCPs than the mixed PFDNCs are listed.
